# Recent Advances in Combination Therapy of YAP Inhibitors with Physical Anti-Cancer Strategies

**DOI:** 10.3390/biom15070945

**Published:** 2025-06-29

**Authors:** Junchi Zhou, Changyan Yu, Wanhong Yang, Nian Jiang, Sanhua Li, Yun Liu, Xinting Zhu

**Affiliations:** 1 The Key Lab of Guizhou Provincial Department of Education for Medical Prevention and Treatment of Tumor, Zunyi Medical University, Zunyi 563000, China; 2 College of Basic Medicine, Zunyi Medical University, Zunyi 563000, China; 3 School of Forensic Medicine, Zunyi Medical University, Zunyi 563000, China

**Keywords:** yes-associated protein, radiation therapy, photothermal therapy, photodynamic therapy, cancer treatment, immunotherapy, combination therapy

## Abstract

In recent years, physical anti-cancer strategies using radiation, light, sound, electricity, and magnetism have shown great potential in cancer treatment. Photodynamic therapy, radiation therapy, photothermal therapy, and other treatments have different advantages. As a critical transcriptional coactivator in the Hippo signaling pathway, Yes-Associated Protein (YAP) is closely related to tumor proliferation, radiation resistance, and immunosuppression. YAP has been a target in immunotherapy, and YAP inhibitors are used in clinical practice. Combining immunotherapy and physical anti-cancer strategies is an anti-cancer program with clinical potential to enhance the therapeutic effect. This review summarizes the role of photodynamic therapy, radiotherapy, and other physical anti-cancer strategies combined with YAP-targeted therapy in cancer treatment. YAP inhibitors and these physical anti-cancer strategies provide new directions and ideas for cancer treatment.

## 1. Introduction

In recent years, physical anti-cancer strategies have made rapid progress and have shown promising therapeutic effects. Photodynamic therapy (PDT) uses photosensitizers (PS) to mediate energy transfer and produce reactive oxygen species (hydroxyl radicals et al.) [1]. Reactive oxygen species (ROS) can damage DNA and protein structures in cells, leading to organelle destruction and cell death. This process can activate the body’s immune system, leading to immunogenic cell death (ICD) [2]. Photothermal therapy can cause local hyperthermia at the tumor site, also leading to tumor cell death and ICD. Ultrasound, magnetic, and electric fields can produce high temperatures. When used in hyperthermia, they can ablate tumors [3,4,5]. Although the principle of radiation therapy is different from that of the above treatments, it can also produce reactive oxygen species to cause ICD. Due to the characteristics of a long treatment cycle and high recurrence rate, radiotherapy is often combined with chemotherapy and immunotherapy to treat tumors [6,7,8,9]. Immunotherapy is also frequently used as an adjunct to other physical anti-cancer strategies. Photodynamic or photothermal therapy, combined with immunotherapy, can enhance the immune response triggered by the ICD [10,11]. Hyperthermia was shown to produce additional stimulatory effects on all the steps of immunotherapy [12].

Yes-Associated Protein (YAP) is located downstream of the Hippo signaling pathway and is the core effector of this pathway. YAP is a potential target for stimulating the patient’s immune system and forming long-term immune memory. YAP promotes immune escape from tumors and tumor microenvironment immunosuppression through the modulation of immune-related factors, such as PD-L1 [13], CD24 [14], CXCL5 [15], etc. In addition to the YAP itself serving as an anti-cancer target, recent studies have also identified YAP-formed nuclear aggregates as potential targets for cancer therapy. YAP, as a transcriptional coactivator, can form nuclear condensates within the cell nucleus. These nuclear condensates play a significant role in tumorigenesis and treatment resistance. In ependymomas (EPN), YAP fusion proteins form membrane-less nuclear condensates through liquid–liquid phase separation (LLPS). These nuclear condensates enrich transcription co-activators (e.g., BRD4, MED1, and TEAD) while excluding inhibitory complexes (e.g., PRC), thereby activating oncogenes (e.g., MYC and Hippo pathway genes) [16]. YAP aggregates have also been shown to have a “super enhancer” effect. They can compartmentalize the YAP transcription factor TEAD1 and other YAP-related coactivators (including TAZ), concentrate epigenetically modified genes (e.g., histone acetyltransferase EP300), and significantly enhance the transcriptional efficiency of oncogenes [17,18]. Therefore, targeting YAP phase-separated aggregates could be a new direction for developing YAP-related targeted anti-cancer inhibitors. YAP inhibitors have demonstrated their anti-cancer effects. Thijs J Hagenbeek et al. identified a potent small molecule inhibitor of YAP, GNE-7883, which showed promising anti-tumor effects in vitro and in vivo in various tumors [19]. However, YAP inhibitors have potential risks to normal human tissues. The inhibition of YAP may affect normal tissue regeneration and repair [20]. More importantly, YAP acts as a tumor suppressor in some tumor-surrounding cells, and it cannot suppress tumors without precisely targeted therapy [19,21]. Changes in drug delivery can improve the targeting of immunosuppressive agents and enhance the therapeutic effect. In recent years, the combination of light, heat, electric field, magnetic field, and other physical means with nanoparticles has made significant progress in delivering immunosuppressive agents [22,23,24,25]. Huang J et al. developed an amphiphilic and block dendritic polymer-based nanoparticle. They applied it to targeted drugs combined with photodynamic therapy for the treatment of drug-resistant non-small cell lung cancer. By combining YAP targeting therapy with physical anti-cancer strategies, the anti-tumor effect can be achieved more efficiently and accurately. It also reduces the potential risks associated with YAP inhibitors. In recent years, several clinical studies of YAP inhibitors for cancer treatment have also been conducted (Table 1).

Regarding the YAP inhibitors, the literature review mainly focused on the mechanism of tumorigenesis, the development of related drugs, and the clinical application prospects in cancer. Previous reviews of physical anti-cancer strategies mostly focused on the material perspective. The review by Saeid Moghassemi summarizes the nanoemulsion formulation and explores the advantages of nanoemulsions for PDT. It also provides a new direction for the development of new photosensitizers [26]. Bin Ji’s review focuses on emerging nanomedicine. It can modulate the immunosuppressive tumor microenvironment (TME) and enhance tumor immunity driven by PDT when combined with photodynamic therapy [10]. Comprehensive discussion on the synergistic effect of YAP inhibitors and physical anti-cancer strategies is limited. This review summarizes the application of the YAP pathway in combined immunotherapy with photodynamic, radiation, and other physical methods in cancer therapy. Information related to the synergistic effects of YAP inhibitors could provide new options for clinical cancer treatment.

## 2. Discussion

### 2.1. YAP and Photodynamic Therapy

Photodynamic therapy (PDT) is a new type of physical anti-cancer strategy formally applied in clinical practice in the last century [27]. Compared with surgery and radiotherapy, it has good clinical efficacy and almost negligible side effects [28]. For example, in 2020, Roderick F.O’Day et al. conducted a clinical trial of PDT for posterior choroidal amelanotic melanomas, with in vivo tumor regression achieved in 88% of patients after treatment [29]. The core of modern photodynamic therapy is light (light source), photosensitizer, and oxygen. PDT relies on the interaction of these three elements to achieve therapeutic effects. After entering the human body, the photosensitizer selectively accumulates in the target tissue. When the target area is irradiated with light of a specific wavelength, the photosensitizer absorbs the light energy and is elevated to an unstable excited state (singlet state) [30]. The short-lived excited singlet state of the photosensitizer may undergo intersystem crossing to form a relatively long-lived triplet state. This long-lived triplet state can generate reactive oxygen species through Type I and Type II reactions. Type II reactions are the primary pathway, where the triplet state directly transfers its energy to the ground-state triplet oxygen, forming excited singlet oxygen. Type I reactions, on the other hand, rely on electron transfer to generate free radicals such as superoxide anion radicals (O_2_^−^) [31,32]. ROS plays a key role in PDT treatment. The reactive oxygen species produced by photosensitizers have strong oxidizing capabilities and can oxidize cell membranes, proteins, nucleic acids, mitochondria, etc., ultimately causing widespread oxidative damage. The cytotoxic cascade reaction caused by ROS can lead to tumor cell death (e.g., necrosis, apoptosis) [33,34]. Tumor cell death also triggers ICD, thereby activating the human immune system, which is one of the mechanisms underlying the long-term efficacy of PDT. Concurrently, the blood vessels surrounding the tumor tissue are also damaged, thereby affecting the destruction of the tumor tissue.

**Figure 1:**
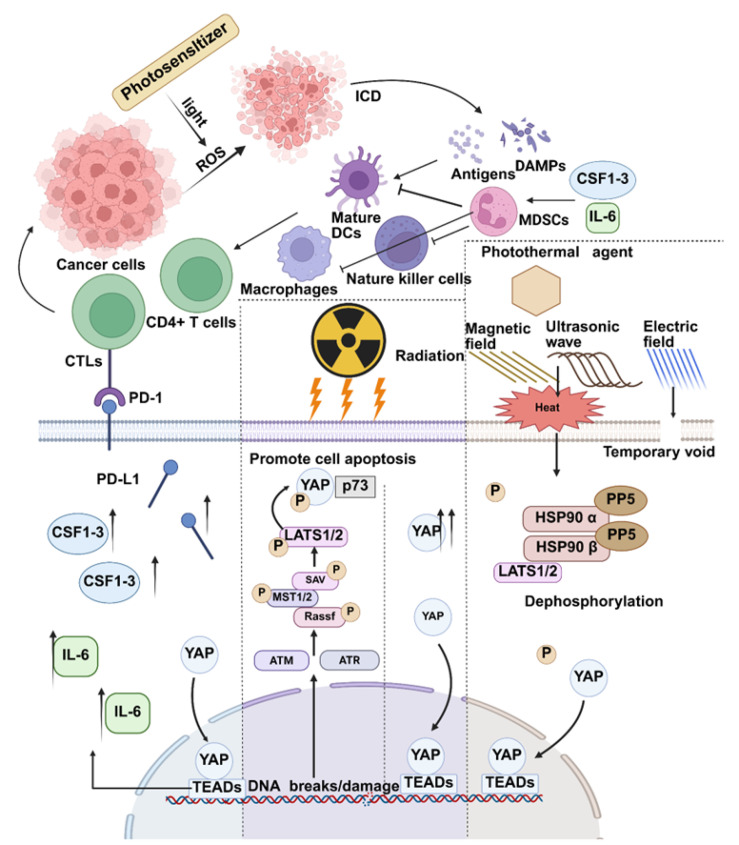
YAP and physical anti-cancer strategies. Photodynamic therapy triggers an ICD with the help of ROS produced, which leads to an increase in the number and maturation of immune cells and an increase in cytotoxic T-cells. This causes a series of stress responses. These include apoptotic transcription of pro-apoptotic genes. In addition to increasing cytoplasmic YAP after the activation of ATM/ATR, it also promotes the formation of a tumor suppressor complex between the remaining nuclear YAP and p73. The high temperature will cause stress in tumor cells, which can develop in response to heat therapy.

In recent years, the development of nanomaterials has brought many possibilities for overcoming the limitations of conventional PS drugs. The combined use of nanomaterials and anti-cancer drugs will improve the therapeutic efficacy and depth of PDT [35], as well as its targeting of tumor tissue [36,37]. For example, Juergen Bartelmess et al. developed a novel pyrene–diiodo-BODIPY dyad and combined it with carbon nano-onions (CNOs) for application. In vitro experiments demonstrated that pyrene–diiodo-BODIPY caused significant light-mediated cytotoxicity and apoptosis in Hela cells [33]. Taotao Liu’s team made a modified copolymer of metronidazole (MN) and RGD peptide with polyacrylic acid (PAA) and polyethylene glycol (PEG). After ultrasonic fracturing, the modified copolymer was coated with liquid metal (LM) nanoparticles to develop RGD-PEG-PAA-MN@LM nanoparticles. This nanoparticle has shown better targeting to liver cancer in vivo. At the same time, the RGD-PEG-PAA-MN@LM nanoparticle can produce more ROS under near-infrared irradiation to achieve a better tumor inhibition effect [38]. While using ROS to kill tumors, combining photodynamic therapy and nanotechnology can also induce immunogenic cell death (ICD) (Figure 1) [39]. Immunogenic cell death is regarded as one of the methods that can achieve complete tumor clearance [2]. Damage-associated molecular patterns (DAMPs) and tumor-associated antigens are released after immunogenic cell death [40]. Immune cells, including natural killer cells, macrophages, dendritic cells (DC), and other related cells, are activated by DAMPs [41]. Then, CD4+ and CD8+T cells were also activated. Ultimately, ICDs induce tumor-specific immunity. However, in photodynamic therapy, there is a state of immunosuppression and immunogenic deficiency in the tumor microenvironment. It reduces the efficiency of photodynamic therapy-induced ICD, and the hypoxic state caused by it further reduces the efficiency. The combination of YAP inhibitors and PDT is considered a viable direction.

**Figure 2:**
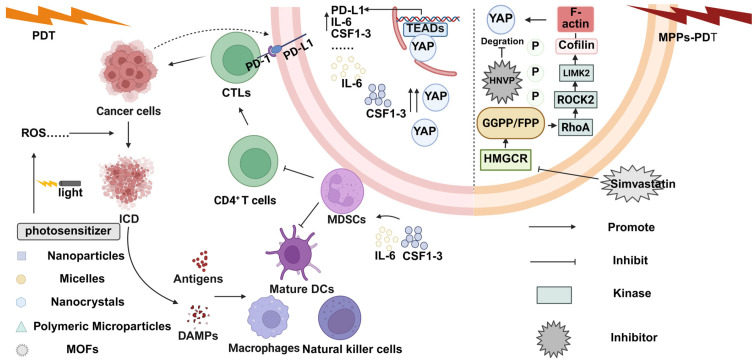
Mechanism of the combination of photodynamic therapy and YAP inhibitors. Photodynamic therapy enhances tumor-specific immunity by triggering ICD. Damage-associated molecular patterns (DAMPs) and tumor-associated antigens are released after immunogenic cell death. After the above events, the body eventually produces tumor-specific immunity and increases cytotoxic T lymphocyte infiltration (CTL). Overexpression of YAP leads to an attenuated ICD-induced immune response, which in turn upregulates the expression of PD-L1, IL-6, and CSF-3 molecules.

YAP is upregulated in many tumors, and it is closely related to the occurrence, proliferation, invasion, drug resistance, and immune escape of tumors [42]. For example, the activation of either YAP or TAZ upregulates PD-L1 (a ligand that transmits immunosuppressive signals) in human breast cancer and promotes tumor immune escape from T cells [43]. In prostate cancer models, YAP activation drives the recruitment of myeloid-derived suppressor cells (MDSCs). MDSCs act on the tumor microenvironment to promote an immunosuppressive tumor microenvironment [44]. The YAP1 protein has also been suggested to promote an immunosuppressive tumor microenvironment by affecting regulatory T cells and macrophages [45]. In addition, Eleni Stampouloglou et al. have also experimentally demonstrated that YAP deficiency led to enhanced T cell activation, differentiation, and function. Therefore, the inhibition of YAP improves the T cell state in the tumor microenvironment [46]. As a second-generation porphyrin photosensitizer, verteporfin (VP) is also a YAP inhibitor. Song M et al. found that the therapeutic efficacy of VP for uveal melanoma (UM) was poor. So, they developed a biocompatible hyaluronic acid nanoparticle (HANP/VP). It showed better targeting and better tumor inhibition in (HANP/VP-mediated photodynamic therapy in animal studies [47]. It also showed that HNVP elicited a stronger immune response. In treating osteosarcoma, the therapeutic resistance encountered with traditional radiotherapy modalities is also unavoidable in photodynamic therapy. (Figure 2) Fangbiao Zhan et al. found that YAP was activated, and nuclear translocation occurred after MPPa-PDT alone on osteosarcoma. Subsequently, they developed the combined use of methyl pyrrole/pyrrole and *α*-methyl ester-mediated photodynamic therapy (MPPa-PDT) targeting the RhoA/ROCK2/LIMK2/YAP pathway (Figure 2). This therapy significantly improves the efficacy of MPPa-PDT in the treatment of osteosarcoma (OS) and increases the sensitivity of the tumor to PDT [48].

Combining YAP inhibitors and photodynamic therapy with monotherapy can improve the immunosuppressive tumor microenvironment (TME) and enhance the anti-tumor effect. PDT has demonstrated its advantages over traditional radiotherapy in tumor treatment. However, it must also eliminate its drawbacks to achieve broader clinical applications. In addition to dealing with the adverse reactions to PDT in clinical practice, it is valuable to solve the problem of oxygen depletion in the tumor microenvironment during treatment, and oxygen-boosted PDT is regarded as one of the approaches [49]. This approach improves immunosuppression in the tumor microenvironment.

### 2.2. YAP and Radiation Therapy

Radiation therapy is a classic cancer treatment method. It is based on the principle of using ionizing radiation of high-energy rays to destroy the DNA of cancer cells and kill them. Since X-rays were first used to treat tumors in the 19th century, radiotherapy has evolved into many types today. The radiation used in modern radiotherapy has expanded from X-rays, alpha rays, and beta rays to neutron beams, proton beams, and so on [50,51]. Based on the previous external radiation therapy, brachytherapy and internal radiation have rapidly developed in recent years with the progress of nanotechnology [52,53]. Therefore, modern radiotherapy protocols can have many variations besides the choice of dose [54,55]. These protocols have many advantages over conventional radiotherapy [56,57]. For example, involved site radiation therapy (ISRT) combines imaging, computer technology, and radiotherapeutic technology. It improves the accuracy of radiotherapy based on the original radiotherapy. ISRT has been applied to the treatment of lymphoma. ISRT is shown to reduce exposure to normal tissues and the risk of toxicity [58]. Ultra-high dose-rate proton therapy (FLASH^pr^) increases the dose and dose rate of original proton therapy. Samriddhi and Shukla et al. compared conventional proton therapy (CONV^pr^) with ultra-high dose-rate proton therapy (FLASH^pr^) in animal experiments. They found that FLASH^pr^ can improve non-small cell lung cancer (NSCLC) cell proliferation, immune rejection, and tumor control [59]. Jean-Pierre Gerard et al. found that neoadjuvant chemoradiotherapy with contact X-ray brachytherapy-enhanced neoadjuvant chemoradiotherapy significantly improves 3-year organ preservation rat es in patients [60].

However, radiotherapy alone still causes side effects. Patients with prostate cancer who receive hyperfractionated stereotactic body radiation therapy (SBRT) experience adverse effects on the digestive system and nervous system [61]. Whole pelvic pencil beam scanning proton radiotherapy (PBS PRT) can be used to treat gynecologic malignancies. However, after treatment, some patients experienced various adverse reactions in the digestive, hematologic, and genitourinary systems. In addition, in recent years, it has been found that radiotherapy also affects the body’s immune system. The finding of the abscopal effect confirms that radiation therapy (RT) can reduce the suppressive effect of regulatory T cells [62,63]. However, researchers have also found that radiotherapy can cause ICD [64]. In the process of radiotherapy, ionizing radiation not only kills tumor cells directly but also indirectly kills tumor cells by producing ROS. This can also lead to ICD [65]. Similarly to what is encountered in photodynamic therapy, immunosuppressive and hypoxic conditions in the tumor microenvironment attenuate ICD effects. Combining radiotherapy with immune-targeted therapy, gene therapy, and other new treatment methods is the future trend to improve radiotherapy’s therapeutic effect.

**Figure 3:**
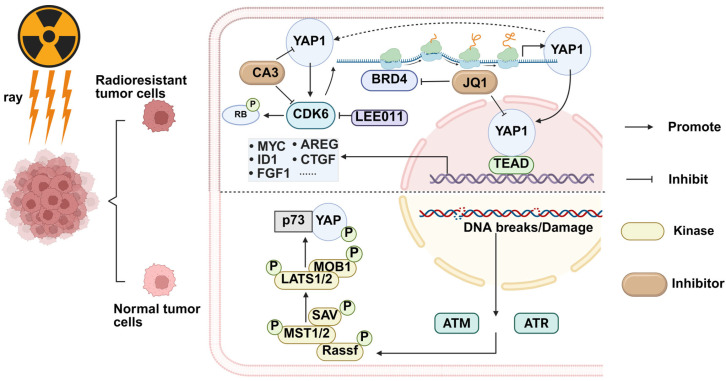
YAP is abnormally elevated in radiotherapy-resistant tumor cells. Combining YAP inhibitors and radiotherapy can alleviate radiotherapy resistance and increase the therapeutic effect. YAP1 overexpression upregulates CDK6 expression and transcription and promotes radiation resistance in drug-resistant esophageal cancer tissues and cell lines. CA3 inhibits both YAP and CDK6, thereby reducing radiation resistance. YAP is activated in normal tumor cells after ionizing radiation, which initiates DNA protection and repair-related pathways.

When DNA in tumor cells is damaged by ionizing radiation, the activation of ataxia telangiectasia-mutated promotes the dimerization of Ras association domain family 1 isoform A (RASSF1A) and its binding to MST2. The activation of LATS1/2 by MST2 increases the amount of YAP in the cytoplasm and promotes the nuclear binding of YAP to p73 as a tumor suppressor complex. At this time, YAP activation leads to apoptosis of tumor cells. However, in many tumors, YAP is abnormally upregulated due to the inactivation of the Hippo pathway. It leads to uncontrolled regulation of tumor stem cell (CSC) activity, cell growth, and proliferation [66] (Figure 3). For example, YAP can induce MCM6 transcription in gastric cancer by maintaining a complex with the small chromosome 6 MCM6 promoter. But MCM6 supports YAP by activating the PI3K/Akt/GSK-3*β* signaling pathway, which overactivates YAP and enhances the occurrence and metastasis of gastric cancer. Kawamoto R et al. found that narcicycline can act as an inhibitor of YAP. It can compete with TEAD4 for binding to YAP, thereby down-regulating YAP overexpression or activation in tumors. Narcicycline has also been shown to inhibit tumor growth in animal experiments [67].

In addition to being associated with cell proliferation activity, YAP also affects tumor migration. Shi C et al. found that knocking down YAP in HCC cells caused mitochondrial dysfunction and ATP deficiency, leading to intracellular calcium overload. It ultimately affected cofilin activity and resulted in HCC migration disorders [68]. At the same time, the activation of YAP also aggravates the immunosuppression in the TME. In conclusion, the combined application of YAP inhibitors and radiotherapy will help attenuate the radiation resistance of tumor cells and enhance the ICD effect. In the study of non-small cell lung cancer, the targeted therapy of YAP also significantly benefits treatment methods such as radiotherapy and chemotherapy. As a key regulator of the Hippo/YAP1 signaling pathway, BRD4 plays an essential role in YAP expression. Shumei Song et al. found that the BRD4 inhibitor JQ1 can be used as a new Hippo/YAP1 signaling pathway inhibitor class. JQ1 mainly targets tumor cells with high YAP1 expression and treatment resistance and can inhibit radioresistant cells with strong cancer stem cell characteristics and invasive phenotype [69]. Fan Li et al. found that YAP1 is overexpressed in tissues and cells of drug-resistant/radiation-resistant esophageal cancer, and CDK6 expression was positively correlated with YAP1. Over-activation of CDK6 can enhance the radiation resistance of tumors. After they treated the resistant esophageal cancer cells with the YAP1 inhibitor CA3, they found that the resistant esophageal cancer cells regained their sensitivity to radiation [70] (Figure 3). It can be concluded that the combination of radiotherapy and YAP-targeted therapy has excellent clinical potential. Cheng H et al. found that the resistance of non-small cell lung cancer cells to cisplatin and ionizing radiation decreased after knocking out the YAP1 gene [71]. Li F et al. combined radiotherapy and an inhibitor that blocks YAP nuclear translocation in radioresistant nasopharyngeal carcinoma (NPC). The combination of radiotherapy and YAP inhibitor resulted in better outcomes than radiotherapy alone [72].

Radiotherapy combined with YAP inhibitor anti-cancer regimens can be improved in many areas. For example, more research is needed on the specific dose, mode, and frequency to achieve the best therapeutic effect in specific regimens for different tumors. Developing safer and more effective YAP inhibitors is critical for future clinical applications.

### 2.3. YAP and Another Therapeutic Method: Hyperthermia and Electrochemotherapy

**Figure 4:**
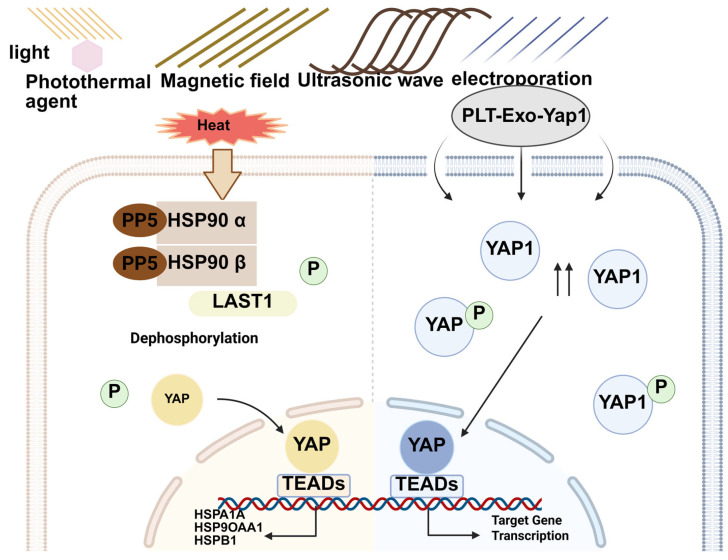
Purified donor platelet-derived exosomes were delivered by electroporation to load recombinant Yap1 protein. YAP and hyperthermia. Light, electric fields, magnetic fields, and ultrasound all produce high temperatures. They can both be used for the hyperthermia of tumors. Tumors can cause heat shock in the face of high heat. HSP90*α*/*β* modulates heat shock-induced dephosphorylation of YAP and LATS1. YAP dephosphorylation is strongly associated with heat shock proteins. YAP enhances the heat shock transcriptome and cell survival.

In recent years, as an emerging physical anti-cancer strategy, hyperthermia has also made rapid progress in clinical practice [73,74,75]. Hyperthermia is a kind of therapy using physical factors (radio frequency, microwave, ultrasound, laser, etc.) to increase the temperature of tumor tissue and/or the whole body and use high temperature to kill tumors [76]. Hyperthermia can also enhance the effect of killing tumors by indirectly affecting the body’s immune system [77]. Hyperthermia can improve immune cell transport by “reversing tumor hypoxia”. Hyperthermia upregulates the expression of active receptors on the surface of natural killer (NK) cells [78]. Photothermal therapy (PTT) is a type of hyperthermia treatment. Its principle involves using a photosensitizer to generate heat under light irradiation of a specific wavelength, which selectively kills tumor cells [79]. By hyperthermia, PTT can kill tumor cells and trigger ICD to promote the body’s immune response to the tumor [80]. Single photothermal therapy cannot deal with metastatic and recurrent tumors, and combining photothermal therapy and immunotherapy can effectively solve this problem [81]. Hyperthermia uses high temperatures to kill tumor cells. However, the thermal effect also promotes the dephosphorylation and activation of YAP. This undoubtedly weakens the therapeutic effect of hyperthermia. Under high-temperature treatment, Min Luo et al. demonstrated that the Hippo pathway plays a vital role in heat shock. YAP and TAZ are activated in heat shock and play an indispensable role in inducing the heat shock transcriptome. Heat shock-induced YAP dephosphorylation is associated with HSP90 [82] (Figure 4). The activation of YAP in thermal response can not only cause heat shock and improve the survival rate of tumor cells but also affect tumor immunity.

Like in PDT, the activation of YAP results in a more substantial lack of immunosuppression and immunogenicity in the tumor microenvironment encountered during PPT. Based on the above, the body’s ICD-induced immune response is attenuated. In addition, some researchers have used the photothermal effect to change the matrix stiffness, thereby changing the phenotype of macrophages. They also confirmed that the ratio of nuclear YAP is positively correlated with the transfer of macrophage phenotype [83]. At present, there is no report on the synergistic treatment of tumors with YAP inhibitors and physical anti-cancer strategies, such as photothermal therapy that uses thermal effects to kill tumors. From the perspective of reducing the survival rate of tumor cells under high temperatures and enhancing tumor immunotherapy, the synergistic treatment of YAP inhibitors and photothermal therapy undoubtedly has clinical potential. The combination of hyperthermia and drugs such as YAP inhibitors can improve the effect of treatment. However, hyperthermia still needs to achieve precise heating of tumor tissues while protecting normal tissues from damage in clinical applications. Therefore, it is vital to control the temperature, time, and location of the heat therapy and to make a personalized heat therapy plan. Applying nanoparticles in thermotherapy can improve this situation by increasing the localization and selectivity of heating. Advances in thermal therapy equipment can also provide greater precision in treatment. For example, image-guided technology and magnetic resonance imaging monitor real-time temperature changes and heating ranges, so that real-time adjustments can be made during treatment [84,85].

Electrochemotherapy (ECT) is another emerging physical anti-cancer strategy. With the combination of electroporation and chemotherapy drugs, electric pulses can make non-permeable drugs enter cells to enhance the toxicity of chemotherapy drugs to tumor cells. ECT has shown promising results in clinical trials for skin-related tumors such as melanoma and Kaposi’s sarcoma (KS) [86,87]. Because electroporation can improve the delivery efficiency of foreign molecules, it has also been used in gene and immunotherapy in recent years [88,89]. Irreversible electroporation therapy is used in electric fields for cancer treatment. This therapy can use high-pressure pulses to create permanent pores in tumor cell membranes [90]. The application of electroporation therapy in combination with YAP inhibitors in cancer treatment has not been reported. However, some malignant tumors of the motor system, such as osteosarcoma, are often treated with surgical resection. Tendon and bone deficits resulting from surgery are significant challenges in the rehabilitation of patients. YAP is important in regulating the proliferative activity of progenitor cells and stem cells, as well as angiogenesis. It is possible that the high delivery efficiency of YAP, combined with electroporation therapy, can be used for rapid recovery after surgical resection of osteosarcoma. Lu J et al. demonstrated that the delivery of purified donor platelet-derived exosomes loaded with recombinant Yap1 protein (PLT-Exo-Yap1) into endogenous tendon stem/progenitor cells (TSPCs) by electroporation could regulate YAP activity. The stemness and differentiation potential of TSPCs were promoted. Afterward, they prepared a GelMA hydrogel with expos-yap1 functionality and parallel array-based matrix structures to enhance tsp adhesion and encourage cell stemness and differentiation of regenerated cells toward tendons for tendon regeneration in vitro and in vivo [91].

Electroporation improves drug delivery efficiency but may cause localized hyperthermia, which can cause thermal damage to the body(Figure 4). Electroporation techniques must be more precise to minimize damage to normal tissues. Therefore, the frequency and intensity of current pulses, electrode materials, and electroporation equipment require improvement and optimization.

## 3. Summary and Outlook

This review demonstrates the clinical potential of YAP inhibitors in combination with the latest physical anti-tumor strategies and insight into their synergistic mechanisms of action. Thanks to advances in nanotechnology, new photosensitizers exhibit enhanced precision and selectivity, thereby improving the therapeutic efficacy of photodynamic therapy (PDT) and reducing side effects. In oncology, PDT has expanded beyond superficial tumors, as endoscopes or optical fibers can now deliver light to deeper tissues to treat deep tumors effectively. However, the limited depth of light penetration in tissues continues to restrict the therapeutic effects of PDT. Factors such as immunosuppression, lack of immunogenicity, and oxygen deficiency in the tumor microenvironment hinder the efficiency of PDT in triggering immunogenic cell death (ICD), which is crucial for PDT’s capacity to kill tumors. Oxygen-enhanced PDT presents one approach to alleviating oxygen deficiency. Further innovation of photosensitizers is necessary to mitigate the potential toxicity of residual photosensitizers in the body. The abnormal upregulation of YAP is closely related to the attenuation of tumor-specific immunity in vivo. The combination of YAP inhibitors and PDT is expected to overcome the obstacles in the process of PDT triggering an immune response and enhance the final efficacy of PDT. Meanwhile, Jie Guo et al. found that YAP inhibitors can also impair tumor DNA repair activity, thereby enhancing the killing of tumor cells by PDT while improving drug resistance in PDT anti-cancer therapy [92]. In addition, YAP inhibitors are expected to enhance the effect of PDT on extracellular matrix (ECM) deposition, thereby enhancing tumor tissue permeability [93,94,95]. If combined with other anti-cancer drugs on this basis, it is expected to enhance its anti-cancer effect. Radiotherapy is now commonly employed with chemotherapy and immunotherapy rather than as a standalone treatment, leading to more efficient and personalized patient care. Nonetheless, radiotherapy remains ineffective against tumors that are less responsive to it, and the aberrant upregulation of YAP is strongly associated with the development of radiotherapy-resistant tumor cells. Research has shown that knocking down YAP or inhibiting its expression can enhance the sensitivity of tumor cells to radiotherapy and improve treatment outcomes [70,96]. This may also relate to YAP’s role in promoting radiotherapy-induced ICD. However, damage to healthy tissues from radiotherapy continues to pose a significant challenge to overcome. Compared to the past, advancements in computer technology have allowed modern radiotherapy protocols to achieve greater precision. Thanks to the development of nanoparticles, contemporary thermotherapy anti-cancer protocols can accurately deliver high temperatures to tumor sites. However, thermotherapy may induce heat shock when utilizing high temperatures to destroy tumors, which can undermine treatment effectiveness. The activation of YAP at elevated temperatures plays a significant role in regulating the heat shock transcriptome, suggesting that the combined use of YAP inhibitors with thermotherapy can enhance its efficacy. In photothermal therapy, YAP inhibitors can similarly aid in the promotion of ICD. In conclusion, YAP inhibitors combined with phototherapy can alleviate the factors affecting the immune response, such as immune deficiency, thereby enhancing the efficiency and intensity of phototherapy-induced ICD. YAP inhibitors can also enhance the direct killing effect of phototherapy on tumors. Despite significant advancements in the clinical application of nanoparticles, evidence of their biosafety remains insufficient.

In summary, despite its drawbacks, we believe that the combination of physical anti-tumor approaches with YAP inhibitors is promising. This review shows the advantages and disadvantages of physical anti-tumor methods, such as PDT-YAP inhibitor combined strategy and radiotherapy–YAP inhibitor combined strategy, combined with YAP inhibitor, hoping to provide broader ideas for tumor treatment. We hope that with the continuous progress of delivery technology and targeted agents, along with the continuous progress of clinical trials, the biological safety issues in the aforementioned combination therapy strategy will be resolved, thereby providing safer and newer treatment options for cancer patients as soon as possible.

## Figures and Tables

**Table 1:** Summary of clinical trials of YAP inhibitors published on the NIH website.

NCT Number	Study Status	Sponsor	Phases	Start Date
NCT06791941	RECRUITING	Regina Elena Cancer Institute		7 March 2024
NCT05228015	TERMINATED	Ikena Oncology	PHASE1	7 January 2022
NCT04857372	RECRUITING	Novartis Pharmaceuticals	PHASE1	21 October 2021

## Data Availability

No new data were created or analyzed in this study. Data sharing is not applicable to this article.
